# Risk factors for return-to-estrus in primiparous Landrace × Yorkshire sows under tropical conditions: A large-scale retrospective study

**DOI:** 10.14202/vetworld.2026.631-641

**Published:** 2026-02-23

**Authors:** Nam Hoai Nguyen, Lanh Thi Kim Do, Thanh Van Nguyen, Dung Van Bui, Dao Tran Anh Bui, Peerapol Sukon

**Affiliations:** 1 Department of Animal Surgery and Theriogenology, Faculty of Veterinary Medicine, Vietnam National University of Agriculture, Hanoi, Vietnam; 2 Department of Veterinary Pathology, Faculty of Veterinary Medicine, Vietnam National University of Agriculture, Hanoi, Vietnam; 3 Faculty of Veterinary Medicine, Khon Kaen University, Thailand. 123 Moo 16 Mittraphap Rd., Nai-Muang, Muang District, Khon Kaen 40002, Thailand; 4 Research Group for Animal Health Technology, Khon Kaen University, Thailand. 123 Moo 16 Mittraphap Rd., Nai-Muang, Muang District, Khon Kaen 40002, Thailand

**Keywords:** age at insemination, Landrace ×, Yorkshire, primiparous sow, reproductive management, return-to-estrus, tropical climate, weaning-to-service interval, sow fertility

## Abstract

**Background and Ai:**

Return-to-estrus (RTE) after insemination is a major source of reproductive inefficiency in commercial swine herds, particularly in primiparous sows that are physiologically distinct from multiparous animals. Under tropical conditions, heat stress, metabolic demands of first lactation, and suboptimal reproductive management may further exacerbate post-weaning fertility problems. However, limited large-scale evidence is available focusing exclusively on first-parity sows. This study aimed to identify reproductive, management, and seasonal risk factors associated with RTE following the first post-weaning insemination in primiparous Landrace × Yorkshire sows raised under tropical conditions.

**Materials and Method:**

A retrospective observational study was conducted using farm records from a commercial herd in central Vietnam. Data from 5,111 primiparous sows were included after applying predefined inclusion and exclusion criteria. Explanatory variables comprised age at first insemination, age at first farrowing, number of piglets born alive (NBA), litter birth weight (LBW), litter size and weight at weaning, lactation length (LL), weaning-to-service interval (WSI), age at first insemination after weaning (ASAI), and month of first post-weaning insemination (MSAI). Univariable and multivariable logistic regression analyses were performed to evaluate associations with RTE. Multicollinearity was assessed using Spearman’s correlation coefficients and variance inflation factors. Model fit and discrimination were evaluated using the Hosmer–Lemeshow test and the area under the receiver operating characteristic curve.

**Result:**

The overall RTE rate was 8.6%. In the multivariable model, MSAI, WSI, ASAI, and NBA were significant predictors of RTE. Sows inseminated during December–May had significantly lower odds of RTE than those inseminated in August–September. A WSI of 2–6 days was associated with the lowest RTE, whereas delayed (7–20 days) intervals increased risk. The lowest RTE was observed in sows with ASAI of 380–400 days. An NBA greater than 14 piglets was associated with a reduced likelihood of RTE. LBW, litter size and weight at weaning, and LL were not independently associated with RTE.

**Conclusio:**

RTE in primiparous sows under tropical conditions is strongly influenced by seasonal timing and post-weaning reproductive management. Optimizing insemination timing, maintaining an appropriate WSI, and mitigating heat stress may substantially improve fertility and reduce non-productive days in tropical swine production systems.

## INTRODUCTION

The return rate, defined as the proportion of sows that return-to-estrus (RTE) after artificial insemination (AI) without establishing pregnancy, is a critical reproductive performance indicator in commercial swine production systems. An elevated return rate reflects unsuccessful conception or early embryonic loss and contributes to prolonged non-productive days, thereby increasing production costs [1]. In practice, the return rate varies substantially with herd management practices, sow parity, environmental conditions, and underlying physiological factors [2-5].

Marked differences in return rate have been reported among countries and production systems. In high-performing herds, return rates as low as 6.6% have been documented, whereas in ordinary herds they may exceed 11.7%–18.5% [6, 7]. In the United States, reported return rates were 15.6% in gilts, 13.9% in parity 1–2 sows, and 11.6% in higher-parity sows [8]. In Brazil, an overall return rate of 10.7% was observed, with particularly high values associated with short lactation length (LL) or large litter size at weaning (LSW) [9]. Similarly, a high return rate of 19.2% across gilts and sows has been reported in Bhutan [10].

Seasonal variation in return rate has been consistently demonstrated. Increased return rates are generally observed during summer and early autumn. For example, return rates were reported to peak in July and August in the Netherlands [11], while higher rates during May–August compared with September–November were observed in the USA [12]. In another study from the United States, Koketsu [7] reported a progressive seasonal increase in return rate, reaching a peak of 17.4% during October–December in ordinary herds.

Parity also plays an important role, with primiparous sows consistently showing higher return rates than multiparous sows [8, 13]. Gilt management, particularly age at first mating, has been identified as a key determinant of return rate. Increasing age at first insemination has been associated with reduced return rate in gilts [14], whereas mating at excessively early or late ages is linked to suboptimal reproductive outcomes [15–17]. In addition, management-related factors such as the weaning-to-service interval (WSI) and LL significantly influence return rate. A WSI exceeding 6 days has been associated with increased return rate [11, 18, 19], and short lactation periods have also been shown to elevate return rate [9, 20].

Despite extensive investigations into RTE in commercial swine herds, important knowledge gaps remain. Most previous studies have evaluated mixed-parity populations, with limited emphasis on primiparous sows, a physiologically distinct group that faces the combined demands of continued growth, first lactation, and post-weaning reproductive recovery. Moreover, key reproductive timing variables specific to first parity, such as age at first AI (AFAI), age at first farrowing (AFF), age at first AI after weaning (ASAI) and month of first post-weaning insemination (MSAI), have rarely been evaluated simultaneously with classical productivity traits, including number of piglets born alive (NBA), litter birth weight (LBW), LSW, litter weight at weaning (LWW), and LL. In addition, evidence derived largely from temperate production systems may not be fully applicable to tropical environments, where chronic heat stress, seasonal fluctuations, and management constraints can substantially alter reproductive responses. The relative contributions of WSI, seasonal insemination timing, and first-parity productivity traits to RTE under tropical conditions therefore remain insufficiently characterized.

The present study aimed to identify and quantify reproductive, management, and seasonal risk factors associated with RTE following the first post-weaning AI in primiparous sows raised under tropical conditions. Specifically, the study evaluated the associations between RTE and AFAI, AFF, NBA, LBW, LSW, LWW, LL, WSI, ASAI, and MSAI using a large-scale retrospective dataset. By focusing exclusively on primiparous sows and integrating variables spanning gilt development, first lactation performance, and post-weaning reproductive management, this study sought to provide evidence-based insights to optimize breeding strategies and reduce RTE in tropical swine production systems.

## MATERIALS AND METHODS

### Ethical approval

This study was conducted exclusively using pre-existing farm management and reproductive records obtained from a commercial Landrace × Yorkshire sow herd located in central Vietnam. No experimental procedures, invasive interventions, or direct animal handling were performed solely for research purposes. All records were anonymized prior to analysis to ensure confidentiality and protection of farm identity.

### Study period, location, and environmental conditions

This retrospective study was conducted from March to July 2025 on a commercial swine farm housing approximately 5,000 Landrace × Yorkshire breeding sows in the central region of Vietnam. The farm is located in a tropical climate zone characterized by persistently high humidity and year-round warm to hot temperatures. The primiparous sows included in the study were born between October 2022 and September 2024, and all post-weaning AI events occurred between November 2023 and May 2025. Monthly mean minimum and maximum ambient temperatures (°C) were as follows: January (15.8–20.4), February (17.1–23.6), March (19.2–25.4), April (25.0–32.9), May (25.1–31.5), June (27.3–34.5), July (27.1–34.0), August (26.8–34.6), September (25.1–32.5), October (22.7–31.4), November (20.8–29.4), and December (15.5–22.1). Monthly relative humidity data were unavailable; however, the annual mean humidity was estimated at 85–86%.

### Husbandry, feeding, and general management

Replacement gilts were group-housed until sexual maturity and subsequently transferred to individual metal crates with fully slatted floors. Both gestation and farrowing units were equipped with ventilation fans and evaporative cooling pads. Automated feeding systems were used throughout all production stages. During the gilt phase, feed allowance was approximately 2.5–2.6 kg/day. During gestation, gilts received 2.2–2.4 kg/day of diets containing 14% crude protein, 0.7% lysine, 8%–9% crude fiber, 7%–8% fat, and 15 MJ/kg metabolizable energy. Lactating sows were fed ad libitum diets with increased protein and lysine contents (16% crude protein, 1.2% lysine). After weaning and until the subsequent AI, sows received 2.5–3.0 kg/day of lactation feed. Water was available ad libitum via nipple drinkers.

Gilts were first bred at a minimum body weight of 135 kg, typically during the second or third estrus. Natural farrowing was allowed, with induction performed on day 116 of gestation using cloprostenol (Hanprost, Hanvet, Hanoi, Vietnam) if spontaneous farrowing did not occur. Dystocia was managed using oxytocin (Dona oxytocin, DonaVet, Dongnai, Vietnam) or manual assistance. All hormonal treatments were administered perivulvarly. Cross-fostering was conducted after the first 24 h postpartum, whereas split suckling was not practiced. Preweaning mortality averaged approximately 7%. Piglets were weaned at a mean age of 25 days, with 11.7% weaned before 21 days of age.

### Reproductive management and estrus detection after weaning

Following weaning, sows received an intramuscular injection of Vitamin AD3E (DonaVet, Dongnai, Vietnam) and were exposed daily to intact Meishan boars for at least 6 h at a ratio of 1 boar per 12 sows. Estrus detection was performed once daily in the early morning using the back pressure test in the presence of a boar. Detection duration ranged from a few seconds to approximately 20 seconds, depending on estrus intensity.

Sows that failed to exhibit estrus within 7 days post-weaning received a second injection of ADE. If estrus was not detected by day 14, alternate-day feed restriction was initiated to stimulate follicular growth. For sows not exhibiting estrus by days 18–21 post-weaning, hormonal treatment with equine chorionic gonadotropin (eCG) and human chorionic gonadotropin (hCG) (Heat 5x, Dong Bang Co. Ltd., Seoul, Korea) was administered intramuscularly in the neck. Boar exposure and photoperiod were subsequently increased to ≥12 h and ≥16 h/day, respectively, to promote estrus expression.

### AI

Sows exhibiting estrus were inseminated using extended semen with a total motility of ≥75% and containing at least 3 × 10^9^ sperm cells per dose. Semen collected from Duroc boars was evaluated microscopically, extended using MR-A® 3 (Kubus, Barcelona, Spain), stored at 15°C, and used for AI within 3 days of collection. Insemination was performed three times in gilts at 8–12 h intervals and twice in sows at 20–24 h intervals.

### RTE determination

Following AI, sows were monitored for RTE from days 18 to 45 post-insemination. Estrus detection was conducted once daily in the morning by trained technicians using a Meishan boar to provide visual and olfactory stimulation. Sows showing behavioral signs of estrus were further examined by stimulation of the vulva, mammary glands, and inguinal area, followed by confirmation of the standing reflex using back pressure.

### Data collection and inclusion and exclusion criteria

A substantial portion of this dataset had been previously used to investigate factors associated with prolonged WSI in primiparous sows [21]. Records from 8,830 animals were initially retrieved. Gilts without AI records, females inseminated but not farrowed, sows that farrowed once but were not inseminated after weaning, sows with fewer than 45 days since AI, and those with incomplete, biologically implausible, or logically inconsistent records were excluded. Ultimately, data from 5,111 primiparous sows were retained for analysis.

### Variable definitions

AFAI and AFF were calculated by subtracting the date of birth from the date of first AI and first farrowing, respectively. NBA was defined as the number of live-born piglets. LBW was calculated as the total weight of live-born piglets with individual birth weights >0.85 kg. LL was defined as the interval from farrowing to weaning. LSW and LWW were defined as the number and total weight of weaned piglets per litter, respectively. WSI was calculated as the interval between weaning and the first post-weaning AI. ASAI was defined as sow’s AFAI after weaning. MSAI corresponded to the calendar month of the first post-weaning AI.

### Classification of independent variables

All continuous variables were categorized prior to analysis to account for nonlinear associations with RTE. AFAI, AFF, NBA, LBW, LSW, LWW, LL, ASAI, WSI, and MSAI were categorized according to predefined biologically relevant intervals without modification.

### Statistical analysis

Statistical analyses were performed using IBM SPSS Statistics for Windows, version 22.0 (IBM Corp., Armonk, NY, USA). A two-step analytical approach was applied. Initially, univariable logistic regression analyses were conducted to evaluate associations between each independent variable and RTE in primiparous sows. Subsequently, all independent variables, regardless of their statistical significance in univariable analyses, were included in a forward multivariable logistic regression model to identify independent predictors of RTE while adjusting for potential confounding effects.

Spearman’s rho correlation coefficients were used to assess collinearity among independent variables, and variables with r ≥ 0.7 were excluded from the same model to avoid multicollinearity. Model fit was evaluated using the Hosmer–Lemeshow goodness-of-fit test. Model performance and parsimony were compared using the Akaike information criterion and the Bayesian information criterion. Multicollinearity among retained variables was further assessed using variance inflation factors. The discriminatory ability of the final model was evaluated using the receiver operating characteristic (ROC) curve, and model accuracy was quantified as the area under the ROC curve (AUC).

## RESULTS

### Descriptive statistics

The overall RTE of the investigated primiparous Landrace × Yorkshire sows was 8.6% (440/5,111) and varied across categories of physiological and management-related factors.

With respect to AFAI, the lowest RTE was observed in the 240–250-day group at 6.5% (38/583), whereas the highest RTE was observed when AFAI exceeded 285 days, at 11.8% (107/909). Sows inseminated before 230 days of age also exhibited a relatively high RTE of 10.7% (56/523), indicating a quadratic relationship between AFAI and reproductive outcome.

A similar pattern was observed for AFF. The lowest RTE (6.5%; 61/941) occurred in sows with AFF of 360–375 days. In contrast, sows that farrowed after 390 days showed higher RTEs (9.7–11.6%), followed by those with AFF below 345 days (10.6%; 49/462), further supporting a quadratic association between AFF and the likelihood of post-insemination RTE.

RTE declined with increasing NBA, from 10.0% (134/1334) in sows with ≤10 piglets to 6.9% (83/1199) in those with >14 piglets. A comparable downward trend was noted for LBW, with RTE decreasing from 11.1% (62/559) in litters <12 kg to 8.3% (152/1,828) in litters >18 kg. For LSW, the lowest RTE was 7.4% (143/1929) in the 13–14 piglet group, whereas the highest RTE was 10.7% (23/214) in litters with >14 piglets. RTEs were broadly comparable across LWW (7.6%–9.3%) and LL categories (7.1%–9.3%).

Marked seasonal variation was observed for MSAI. The lowest RTE occurred in April–May (4.9%; 37/755), followed by December–January (6.0%; 68/1,126). In contrast, higher RTEs were recorded during June–July (13.5%; 18/133), August–September (16.7%; 47/281), and October–November (12.0%; 116/965). Regarding ASAI, the lowest RTE was observed at 380–400 days (5.5%; 52/954), whereas higher RTEs were recorded in sows with ASAI <380 days (10.6%–13.0%) and >430 days (10.8%–11.8%).

The lowest RTE by WSI category was observed in sows inseminated 2–6 days after weaning (7.3%; 234/3210), followed by those with WSI >20 days (7.7%; 64/835). In contrast, higher RTEs were recorded in sows with very short (0–1 days: 13.4%; 9/67) or prolonged WSI (7–8 days: 12.9%; 77/597; 9–10 days: 17.6%; 25/142; 11–20 days: 11.9%; 31/260), indicating a nonlinear relationship between WSI and RTE.

### Univariate analysis

Univariate logistic regression analysis identified significant associations between post-weaning RTE and AFAI, AFF, NBA, LBW, MSAI, ASAI, and WSI. No significant associations were observed for LSW, LWW, or LL (Table 1).

**Table 1:** Univariate analysis of potential factors for return rate in primiparous Landrace x Yorkshire sows.

Factors	Return rate (%)	OR (95% CI)	p-value
Age at the first AI			
189–230 days	10.71 (56/523)	1.72 (1.12–2.64)	0.013
230–240 days	8.07 (36/446)	1.26 (0.78–2.02)	0.340
240–250 days	6.52 (38/583)	reference	
250–265 days	7.59 (91/1199)	1.18 (0.80–1.74)	0.413
265–285 days	7.72 (112/1451)	1.20 (0.82–1.76)	0.349
>285 days	11.77 (107/909)	1.91 (1.30–2.81)	0.001
Age at the first farrowing			
302–345 days	10.61 (49/462)	1.71 (1.16–2.54)	0.007
345–360 days	7.91 (50/632)	1.24 (0.84–1.83)	0.279
360–375 days	6.48 (61/941)	reference	
375–390 days	7.00 (87/1243)	1.09 (0.77–1.52)	0.634
390–410 days	9.73 (104/1069)	1.56 (1.12–2.16)	0.009
>410 days	11.65 (89/764)	1.90 (1.35–2.68)	<0.001
Number of alive piglets born at first parity			
1–10 piglets	10.05 (134/1334)	reference	
11–12 piglets	8.59 (55/640)	0.84 (0.61–1.17)	0.305
13–14 piglets	8.67 (168/1938)	0.85 (0.67–1.08)	0.182
>14 piglets	6.92 (83/1199)	0.67 (0.50–0.89)	0.005
Litter birth weight at the first parity			
<12 kg	11.09 (62/559)	reference	
12–15 kg	8.76 (85/970)	0.77 (0.55–1.09)	0.138
15–18 kg	8.04 (141/1754)	0.70 (0.51–0.96)	0.027
>18 kg	8.31 (152/1828)	0.73 (0.53–0.99)	0.045
Litter size at the first weaning			
<11 piglets	9.02 (56/621)	0.82 (0.49–1.37)	0.456
11–12 piglets	9.30 (217/2335)	0.85 (0.54–1.34)	0.486
13–14 piglets	7.41 (143/1929)	0.67 (0.42–1.06)	0.085
>14 piglets	10.75 (23/214)	reference	
Litter weight at the first weaning			
<70 kg	8.92 (66/740)	reference	
70–80 kg	9.27 (128/1381)	1.04 (0.76–1.42)	0.79
80–90 kg	8.81 (147/1668)	0.99 (0.73–1.34)	0.929
>90 kg	7.63 (96/1258)	0.84 (0.61–1.17)	0.309
First lactation length			
<22 days	9.02 (54/599)	1.30 (0.85–1.98)	0.221
22–24 days	7.85 (109/1389)	1.12 (0.77–1.62)	0.557
25–28 days	9.29 (235/2530)	1.34 (0.96–1.89)	0.090
>28 days	7.08 (42/593)	reference	
Month of the first AI after the first weaning			
December–January	6.04 (68/1126)	0.32 (0.22–0.48)	<0.001
February–March	8.32 (154/1851)	0.45 (0.32–0.64)	<0.001
April–May	4.90 (37/755)	0.26 (0.16–0.41)	<0.001
June–July	13.53 (18/133)	0.78 (0.43–1.40)	0.405
August–September	16.73 (47/281)	reference	
October–November	12.02 (116/965)	0.68 (0.47–0.98)	0.040
Age at first AI after first weaning			
332–370 days	12.97 (41/316)	2.59 (1.68–3.98)	<0.001
370–380 days	10.56 (30/284)	2.05 (1.28–3.28)	0.003
380–400 days	5.45 (52/954)	reference	
400–430 days	7.05 (142/2014)	1.32 (0.95–1.83)	0.100
430–450 days	11.81 (100/847)	2.32 (1.64–3.29)	<0.001
>450 days	10.78 (75/696)	2.10 (1.45–3.03)	<0.001
Weaning to the first AI interval			
2–6 days	7.29 (234/3210)	reference	
0–1 days	13.43 (9/67)	1.97 (0.97–4.03)	0.062
7–8 days	12.90 (77/597)	1.88 (1.43–2.48)	<0.001
9–10 days	17.61 (25/142)	2.72 (1.73–4.27)	<0.001
11–20 days	11.92 (31/260)	1.72 (1.16–2.56)	0.007
>20 days	7.66 (64/835)	1.06 (0.79–1.41)	0.712

OR = Odds ratio, 95% CI = 95% confidence interval.

### Multivariate analysis

Multivariate logistic regression analysis showed that MSAI, WSI, ASAI, and NBA were the most important predictors of RTE in primiparous sows (Table 2). Because of strong correlations with ASAI, AFAI and AFF were excluded from the final model (Spearman’s rho >0.7). The model explained a modest proportion of the variance (Nagelkerke R² = 5.2%) and demonstrated good fit, as indicated by a non-significant Hosmer–Lemeshow test (p = 0.915). Variance inflation factor values ranged from 1.008 to 1.158, indicating no multicollinearity. Model discrimination was fair, with an AUC of 0.655.

**Table 2:** Multivariate analysis of risk factors for return rate in primiparous Landrace x Yorkshire sows.

Factors	OR (95% CI)	p-value
Number of piglets born alive at first parity		
1–10 piglets	Reference	–
11–12 piglets	0.82 (0.59–1.15)	0.252
13–14 piglets	0.83 (0.65–1.06)	0.135
>14 piglets	0.66 (0.49–0.88)	0.005
Month of first AI after weaning		
December–January	0.47 (0.30–0.74)	0.001
February–March	0.57 (0.38–0.84)	0.005
April–May	0.30 (0.19–0.48)	<0.001
June–July	0.65 (0.34–1.25)	0.200
August–September	Reference	–
October–November	0.74 (0.47–1.17)	0.197
Age at first AI after weaning		
332–370 days	1.84 (1.15–2.94)	0.012
370–380 days	1.76 (1.08–2.87)	0.023
380–400 days	Reference	–
400–430 days	1.41 (0.99–2.01)	0.060
430–450 days	2.12 (1.42–3.14)	<0.001
>450 days	2.15 (1.37–3.38)	0.001
Weaning-to-first AI interval		
2–6 days	Reference	–
0–1 days	1.73 (0.84–3.57)	0.140
7–8 days	1.77 (1.33–2.34)	<0.001
9–10 days	2.25 (1.41–3.60)	<0.001
11–20 days	1.63 (1.08–2.46)	0.020
>20 days	0.90 (0.65–1.26)	0.549

OR = Odds ratio, 95% CI = 95% confidence interval.

Compared with sows with NBA of 1–10 piglets, those with 11–12 and 13–14 piglets showed no significant difference in RTE (p > 0.05). However, sows with >14 piglets exhibited a significantly lower RTE (Odds ratio [OR] = 0.66, 95% confidence interval [CI] = 0.49–0.88, p = 0.005).

MSAI exerted a pronounced effect on RTE. Relative to sows inseminated in August–September, those inseminated in December–January (OR = 0.47, 95% CI = 0.30–0.74, p = 0.001), February–March (OR = 0.57, 95% CI = 0.38–0.84, p = 0.005), and April–May (OR = 0.30, 95% CI = 0.19–0.48, p < 0.001) had significantly lower odds of RTE. No significant differences were observed for June–July or October–November (p > 0.05).

Using ASAI of 380–400 days as the reference, sows with ASAI of 332–370 days (OR = 1.84, 95% CI = 1.15–2.94, p = 0.012) and 370–380 days (OR = 1.76, 95% CI = 1.08–2.87, p = 0.023) showed moderately increased odds of RTE. Substantially higher odds were observed in sows with ASAI of 430–450 days (OR = 2.12, 95% CI = 1.42–3.14, p < 0.001) and >450 days (OR = 2.15, 95% CI = 1.37–3.38, p = 0.001).

Relative to sows inseminated 2–6 days post-weaning, those inseminated at 7–8 days (OR = 1.77, 95% CI = 1.33–2.34, p < 0.001) and 11–20 days (OR = 1.63, 95% CI = 1.08–2.46, p = 0.020) had moderately higher odds of RTE, whereas sows inseminated at 9–10 days showed a marked increase (OR = 2.25, 95% CI = 1.41–3.60, p < 0.001). No significant differences were observed for sows inseminated at 0–1 days or >20 days after weaning (p > 0.05), which may be partly attributable to the small number of sows with very short WSI.

## DISCUSSION

### Comparison with previous studies

In the present study, the RTE of primiparous sows (8.6%) was slightly lower than values reported in several previous investigations, many of which included mixed-parity populations. Peltoniemi et al. [22] reported an RTE of 10.4% in Finnish herds, whereas Koketsu et al. [7] documented an RTE of 11.6% in high-performing herds in the United States. Other studies from the United States reported RTE values of 13.9% for parity 1–2 sows [8] and 10.1% for primiparous sows [20]. Similarly, Vargas et al. [9] observed an RTE of 10.7% in Brazilian herds. In Spanish, Portuguese, and Italian herds, the RTE in primiparous sows was reported to be 12.3% [13]. Overall, the RTE observed in this study is broadly consistent with previously published data, indicating that reproductive performance of primiparous sows in the present herd was within expected ranges.

### Effects of WSI on RTE

The association between WSI and RTE observed in this study is consistent with earlier findings. Higher farrowing rates have been reported in sows with a WSI of 0–6 days compared with those with 7–20 days [19, 23]. Likewise, improved farrowing rates were observed in sows with WSI of 3–5 days compared with 0–2 and 9–12 days [24], and in sows with WSI of 4–6 days compared with 7–20 days [25]. The increased RTE associated with WSI of 7–20 days may be explained by impaired follicular development and altered metabolic status. Longer WSI has been linked to smaller follicle size at weaning and reduced litter size, indicating compromised follicular quality and ovulation capacity [26]. Such sows are also more likely to exhibit shortened estrus and estrus-to-ovulation intervals, increasing the risk of suboptimal insemination timing [27]. In addition, greater protein and body weight losses during lactation are associated with poorer follicular quality [28] and prolonged WSI [29]. These metabolic deficits may delay post-weaning ovarian recovery and impair embryonic development and survival, particularly in sows losing more than 5% of body weight [30, 31]. Consequently, a WSI of 7–20 days likely reflects inadequate physiological readiness for breeding and contributes to increased RTE. In contrast, when WSI exceeds 20 days, reproductive function may have sufficient time to recover [29], resulting in RTE levels comparable to those observed during the early post-weaning period. Conversely, the high RTE observed in sows with WSI of 0–1 days may be related to suboptimal follicular development [25] and an increased risk of ovarian cyst formation [32].

### Effects of MSAI on RTE

The seasonal pattern identified in this study aligns with previous reports demonstrating reduced fertility from summer to late autumn. Higher RTE or lower farrowing rates during warmer months have been documented in herds from the United States, Spain, Finland, and Japan [7, 12, 13, 22, 33]. In contrast, studies conducted in Thailand and Nigeria reported comparable or even higher farrowing rates during hot seasons [34, 35]. The elevated RTE observed between June and November compared with December and May may be attributed to increased ambient temperatures. Maximum temperatures occurring 2–3 weeks before AI exert the strongest negative effect on farrowing rate [36, 37]. High ambient temperatures reduce feed intake in lactating sows [38, 39], resulting in increased body weight loss, which has been linked to impaired follicular development [40, 41]. Moreover, heat stress induces hormonal disturbances during early pregnancy [42–44]. Collectively, these factors may explain the higher RTE in sows inseminated from June to November compared with December to May. The higher RTE observed in October–November compared with April–May remains unclear, as ambient temperatures are typically higher in April–May. One possible explanation is the cumulative negative effect of prolonged heat exposure during the preceding summer and autumn, whereas sows inseminated in April–May may have benefited from recovery during cooler months from December to March.

### Effects of AFAI, AFF, and ASAI on RTE

This study identified optimal timing windows for key reproductive indicators, with AFAI of 240–250 days, AFF of 360–375 days, and ASAI of 380–400 days being associated with the lowest RTE. Bertoldo et al. [15] reported increased risks of pregnancy loss when AFAI was either <200 or >400 days. Similarly, Iida et al. [36] observed lower farrowing rates in gilts and sows with AFAI between 269 and 400 days compared with those inseminated before 269 days, and Koketsu et al. [17] demonstrated reduced gilt farrowing rates with increasing AFAI. In contrast, Tummaruk et al. [14] reported a decrease in RTE with increasing AFAI, while Holm et al. [16] found higher RTE in primiparous sows with lower AFAI. These discrepancies may reflect differences in the AFAI ranges evaluated, as Tummaruk et al. [14] included gilts with AFAI ≥150 days, whereas Holm et al. [16] applied a threshold of ≥120 days. Collectively, these findings provide practical guidance for optimizing insemination timing to reduce reproductive failure.

### Effect of NBA on RTE

The inverse association between NBA and RTE observed in this study contrasts with the findings of Tummaruk et al. [14], who reported no significant effect of NBA on subsequent RTE. One possible explanation is that NBA reflects overall reproductive efficiency, encompassing ovulation rate, fertilization success, embryo quality, and implantation capacity. Higher NBA may therefore identify sows with superior reproductive potential, which are more likely to sustain pregnancy following subsequent AI, resulting in lower RTE.

### Lack of effects of LBW, LSW, LWW, and LL on RTE

After adjustment for other variables, LBW, LSW, LWW, and LL were not significantly associated with RTE. This is consistent with previous reports. Hidalgo et al. [45] found no difference in farrowing rate between sows with LLs of 3 and 5 weeks. Similarly, Arend et al. [46] reported no difference in pregnancy or farrowing rates between primiparous sows nursing 12 piglets and those nursing 15–16 piglets, supporting the present finding for LSW. In contrast, Vargas et al. [9] reported higher RTE in sows weaning 12 piglets compared with those weaning 7–11 piglets and observed the highest RTE in sows with LLs of 15–19 days. Koketsu et al. [33] also reported reduced farrowing rates in sows with shorter LLs (8–19 days) compared with longer durations (20–28 days). Although LL, LSW, and LWW may contribute to depletion of sow body reserves during lactation, their effects on RTE appear to be indirect and mediated through WSI [21]. Likewise, LBW does not directly reflect ovarian function to the same extent as NBA. Because WSI and NBA were retained in the multivariate model, the effects of LL, LSW, LWW, and LBW were no longer statistically significant, indicating that their influence on RTE is weaker and mediated through more proximal determinants.

### Limitations

Several limitations of this study should be acknowledged. First, all data were derived from a single commercial herd managed under tropical conditions; therefore, the findings may not be directly generalizable to herds operating in different climatic regions, management systems, or production settings. Second, the study population comprised a single genetic line, which restricts extrapolation of the results to herds using other genotypes.

Third, several key physiological and management-related variables known to influence reproductive performance were not available, including body condition score at weaning, lactation feed intake, postpartum health status, and sow body weight loss. The absence of these potential confounders may have influenced the observed associations, particularly in the context of tropical heat stress. Fourth, the lack of regional humidity data precluded calculation of the temperature–humidity index, thereby limiting a more precise evaluation of heat stress effects.

Fifth, RTE could not be categorized into regular, irregular, or late return patterns because such information was not recorded by the farm, potentially obscuring biologically relevant differences in underlying reproductive mechanisms. In addition, the exclusive focus on primiparous sows limits the applicability of these findings to multiparous animals. Finally, as this was a retrospective observational study based on routinely collected farm records, causal inferences cannot be drawn from the observed associations.

## CONCLUSION

This large-scale retrospective study demonstrated that RTE in primiparous Landrace × Yorkshire sows under tropical conditions was 8.6%, a value comparable to or slightly lower than those reported in previous studies. Multivariate analysis identified MSAI, WSI, ASAI, and NBA as the most influential predictors of RTE. Specifically, lower RTE was observed in sows inseminated during December–May, with an optimal WSI of 2–6 days, an ASAI of 380–400 days, and an NBA >14 piglets. In contrast, reproductive traits such as LBW, LSW, LWW, and LL were not independently associated with RTE after adjustment for confounding factors, indicating that post-weaning reproductive timing and physiological readiness play a more critical role than litter performance traits alone.

These findings provide clear, evidence-based guidance for improving reproductive efficiency in primiparous sows under tropical conditions. From a management perspective, maintaining a WSI of 2–6 days, avoiding excessively early or delayed ASAI, and strategically scheduling AI during cooler months may substantially reduce RTE. In addition, the association between higher NBA and reduced RTE suggests that first-parity performance can serve as a useful indicator for identifying sows with superior subsequent reproductive potential. Collectively, these strategies may help reduce non-productive days, improve herd fertility, and enhance overall productivity in tropical swine production systems.

A major strength of this study is the large sample size derived from a well-documented commercial herd, allowing robust statistical analysis focused exclusively on primiparous sows. The integration of variables spanning gilt development, first-parity performance, seasonal effects, and post-weaning reproductive management provides a comprehensive evaluation of RTE determinants in a physiologically distinct group often under-represented in previous research.

Future studies should incorporate additional physiological and management variables, including body condition score at weaning, lactation feed intake, sow weight loss, postpartum health disorders, and temperature–humidity index, to further elucidate mechanisms underlying RTE. Expanding analyses to multiple herds, diverse genotypes, and multiparous sows would improve generalizability. Prospective or intervention-based studies evaluating targeted management adjustments may also help establish causal relationships.

Overall, this study highlights the central role of post-weaning reproductive timing, seasonal conditions, and first-parity performance in determining RTE in primiparous sows. Optimizing AI timing and mitigating environmental and metabolic stressors appear to be more effective strategies for reducing RTE than relying solely on traditional litter performance indicators. These findings contribute valuable insights for refining reproductive management practices in tropical swine production systems.

## DATA AVAILABILITY

The supplementary data can be made available from the corresponding author upon request.

## AUTHORS’ CONTRIBUTIONS

NHN, DTKL, NVT, BVD, BTAD, and PS: Conceived and designed the study. NHN: Collected data. NHN and PS: Analyzed data, interpreted the results, and drafted and revised the manuscript. All the authors participated in scientific discussion, read, reviewed, and approved the final manuscript.

## COMPETING INTERESTS

The authors declare that they have no competing interests.

## PUBLISHER’S NOTE

Veterinary World remains neutral with regard to jurisdictional claims in the published institutional affiliations.

## References

[1] Koketsu Y, Iida R (2020). Farm data analysis for lifetime performance components of sows and their predictors in breeding herds. Porcine Health Manag.

[2] Shipman GL, Rosero D, van Heugten E (2025). Supplementation of high levels of essential fatty acids using soybean oil in lactation diets benefits the subsequent reproduction of sows but can be detrimental to the performance of young sows if provided after weaning. J Anim Sci Biotechnol.

[3] Sanz-Fernández S, Díaz-Gaona C, Simões J, Casas-Rosal JC, Alòs N, Tusell L, Quintanilla R, Rodríguez-Estévez V (2024). The impact of herd age structure on the performance of commercial sow-breeding farms. Porcine Health Manag.

[4] Crespo S, Gadea J (2024). Use of a vaginally administered gel containing the GnRH agonist triptorelin and a single fixed-time artificial insemination in pigs under commercial conditions:Productive and economic impacts. Animals.

[5] Capoferri R, Parati K, Puglisi R, Moscati L, Sensi M, Lombardi G, Sandri G, Briani C, Galli A (2021). Comparison between single- and group-housed pregnant sows for direct and indirect physiological, reproductive, welfare indicators and gene expression profiling. J Appl Anim Welf Sci.

[6] Savic R, Marcos RA, Petrovic M, Radojkovic D, Radovic C, Gogic M (2017). Fertility of boars –what is important to know. Biotechnol Anim Husb.

[7] Koketsu Y (2000). Productivity characteristics of high-performing commercial swine breeding farms. J Am Vet Med Assoc.

[8] Koketsu Y (2003). Re-serviced females on commercial swine breeding farms. J Vet Med Sci.

[9] Vargas AJ, Bernardi ML, Bortolozzo FP, Mellagi APG, Wentz I (2009). Factors associated with return-to-estrus in first service swine females. Prev Vet Med.

[10] Tsheten G, Penjor T (2024). Determinants of repeat breeding in sows and gilts at the National Piggery Development Centre in Bhutan:A retrospective study. Bhutan J Anim Sci.

[11] Elbers AR, van Rossem H, Schukken YH, Martin SW, van Exsel AC, Friendship RM, Tielen MJ (1994). Return to oestrus after first insemination in sow herds (incidence, seasonality, and association with reproductivity and some blood parameters). Vet Q.

[12] Arend LS, Minton AM, Schwab CR, Shull CM, Buysse SG, Johnston ME, Roaten CM, Anderson CL, Webel SK, Knox RV (2025). The effect of ovulation synchronization with OvuGel on ovarian follicles and fertility responses to a single fixed-time insemination in different parities and seasons. Transl Anim Sci.

[13] Tani S, Piñeiro C, Koketsu Y (2016). Recurrence patterns and factors associated with regular, irregular, and late return to service of female pigs and their lifetime performance on southern European farms. J Anim Sci.

[14] Tummaruk P, Lundeheim N, Einarsson S, Dalin AM (2001). Effect of birth litter size, birth parity number, growth rate, backfat thickness and age at first mating of gilts on their reproductive performance as sows. Anim Reprod Sci.

[15] Bertoldo M, Grupen CG, Thomson PC, Evans G, Holyoake PK (2009). Identification of sow-specific risk factors for late pregnancy loss during the seasonal infertility period in pigs. Theriogenology.

[16] Holm B, Bakken M, Vangen O, Rekaya R (2005). Genetic analysis of age at first service, return rate, litter size, and weaning-to-first service interval of gilts and sows. J Anim Sci.

[17] Koketsu Y, Takahashi H, Akachi K (1999). Longevity, lifetime pig production and productivity, and age at first conception in a cohort of gilts observed over six years on commercial farms. J Vet Med Sci.

[18] Kaneko M, Iida R, Koketsu Y (2013). Herd management procedures and factors associated with low farrowing rate of female pigs in Japanese commercial herds. Prev Vet Med.

[19] Tummaruk P, Tantasuparuk W, Techakumphu M, Kunavongkrit A (2010). Influence of repeat-service and weaning-to-first-service interval on farrowing proportion of gilts and sows. Prev Vet Med.

[20] Koketsu Y, Dial GD, King VL (1997). Returns to service after mating and removal of sows for reproductive reasons from commercial swine farms. Theriogenology.

[21] Nam NH, Lanh DTK, Thanh NV, Dung BV, Sukon P (2025). Determinants of prolonged weaning-to-service interval in primiparous Landrace ×Yorkshire sows under tropical conditions. Vet World.

[22] Peltoniemi OA, Heinonen M, Leppävuori A, Love RJ (1999). Seasonal effects on reproduction in the domestic sow in Finland –a herd record study. Acta Vet Scand.

[23] Sasaki Y, Fujie M, Nakatake S, Kawabata T (2018). Quantitative assessment of the effects of outside temperature on farrowing rate in gilts and sows by using a multivariate logistic regression model. Anim Sci J.

[24] Poleze E, Bernardi ML, Amaral Filha WS, Wentz I, Bortolozzo FP (2006). Consequences of variation in weaning-to-estrus interval on reproductive performance of swine females. Livest Sci.

[25] Hoshino Y, Koketsu Y (2008). A repeatability assessment of sows mated 4–6 days after weaning in breeding herds. Anim Reprod Sci.

[26] Lopes TP, Padilla L, Bolarin A, Rodriguez-Martinez H, Roca J (2020). Ovarian follicle growth during lactation determines the reproductive performance of weaned sows. Animals.

[27] Kemp B, Soede NM (1996). Relationship of weaning-to-estrus interval to timing of ovulation and fertilization in sows. J Anim Sci.

[28] Clowes EJ, Aherne FX, Foxcroft GR, Baracos VE (2003). Selective protein loss in lactating sows is associated with reduced litter growth and ovarian function. J Anim Sci.

[29] Tantasuparuk W, Lundeheim N, Dalin AM, Kunavongkrit A, Einarsson S (2000). Effects of lactation length and weaning-to-service interval on subsequent farrowing rate and litter size in Landrace and Yorkshire sows in Thailand. Theriogenology.

[30] Weaver AC, Kind KL, Kelly JM, Herde P, van Wettere WHEJ (2024). Effect of split weaning on follicle development and oocyte quality in multiparous sows. Anim Reprod Sci.

[31] Weaver AC, Kind KL, Herde PJ, van Wettere WHEJ (2024). Split weaning improves pregnancy rate and embryo survival in sows mated in lactation. Anim Reprod Sci.

[32] Castagna CD, Peixoto CH, Bortolozzo FP, Wentz I, Neto GB, Ruschel F (2004). Ovarian cysts and their consequences on the reproductive performance of swine herds. Anim Reprod Sci.

[33] Koketsu Y, Dial GD, King VL (1997). Influence of various factors on farrowing rate on farms using early weaning. J Anim Sci.

[34] Pearodwong P, Tretipskul C, Panyathong R, Sang-Gassanee K, Collell M, Muns R, Tummaruk P (2020). Reproductive performance of weaned sows after single fixed-time artificial insemination under a tropical climate. Theriogenology.

[35] Hagan JK, Etim NN (2019). The effects of breed, season and parity on the reproductive performance of pigs reared under hot and humid environments. Trop Anim Health Prod.

[36] Iida R, Piñeiro C, Koketsu Y (2021). Timing and temperature thresholds of heat stress effects on fertility performance of different parity sows in Spanish herds. J Anim Sci.

[37] Bloemhof S, Mathur PK, Knol EF, van der Waaij EH (2013). Effect of daily environmental temperature on farrowing rate and total born in dam line sows. J Anim Sci.

[38] Tummaruk P, De Rensis F, Kirkwood RN (2023). Managing prolific sows in tropical environments. Mol Reprod Dev.

[39] Dourmad JY, Le Velly V, Gourdine JL, Renaudeau D (2022). Effect of ambient temperature in lactating sows:A meta-analysis and simulation approach in the context of climate change. Anim Open Space.

[40] Liu K, Zhang L, Xu X, Song M, Ding H, Xiao L, Wen J, Zhou C, Bai J, Liu Y (2025). Lactational high weight loss impairs follicular development by causing mitochondrial dysfunction of ovarian cells in sows and mitigated by butyrate supplement. J Adv Res.

[41] Yu Q, Teerds KJ, Keijer J, Soede NM (2024). Lactation affects post-weaning metabolic profiles, but not follicle size in multiparous sows. Animal.

[42] Kim HD, Kim SH, Lee SY, Kim TG, Heo SE, Seo YR, Cho JK, Jang M, Yun SH, Kim SJ, Lee WJ (2023). Heat stress during summer reduced the ovarian aromatase expression of sows in Korea. Korean J Vet Serv.

[43] Kim HD, Kim YJ, Jang M, Bae SG, Yun SH, Lee MR, Seo YR, Cho JK, Kim SJ, Lee WJ (2022). Heat stress during summer attenuates expression of the hypothalamic kisspeptin, an upstream regulator of the hypothalamic-pituitary-gonadal axis, in domestic sows. Animals (Basel).

[44] Kim TG, Lee HS, Kim SH, Son YB, Heo SE, Kim DY, Kim SJ, Lee WJ (2025). Heat stress-induced CYP1A2 upregulation in sow ovarian granulosa cells. J Anim Reprod Biotechnol.

[45] Hidalgo DM, Friendship RM, Greiner L, Manjarin R, Amezcua MR, Dominguez JC, Kirkwood RN (2014). Influence of lactation length and gonadotrophins administered at weaning on fertility of primiparous sows. Anim Reprod Sci.

[46] Arend LS, Vinas RF, Silva GS, Lower AJ, Connor JF, Knox RV (2023). Effects of nursing a large litter and ovarian response to gonadotropins at weaning on subsequent fertility in first parity sows. J Anim Sci.

